# Arachidonic acid promotes skin wound healing through induction of human MSC migration by MT3-MMP-mediated fibronectin degradation

**DOI:** 10.1038/cddis.2015.114

**Published:** 2015-05-07

**Authors:** S Y Oh, S-J Lee, Y H Jung, H J Lee, H J Han

**Affiliations:** 1 Department of Veterinary Physiology, College of Veterinary Medicine, Research Institute for Veterinary Science, and BK21 PLUS Creative Veterinary Research Center, Seoul National University, Seoul, 151-741, Korea

## Abstract

Arachidonic acid (AA) is largely released during injury, but it has not been fully studied yet how AA modulates wound repair with stem cells. Therefore, we investigated skin wound-healing effect of AA-stimulated human umbilical cord blood-derived mesenchymal stem cells (hUCB-MSCs) *in vivo* and its molecular mechanism *in vitro*. We found that transplantation of hUCB-MSCs pre-treated with AA enhanced wound filling, re-epithelization, and angiogenesis in a mouse skin excisional wound model. AA significantly promoted hUCB-MSCs migration after a 24 h incubation, which was inhibited by the knockdown of G-protein-coupled receptor 40 (GPR40). AA activated mammalian target of rapamycin complex 2 (mTORC2) and Akt^ser473^ through the GPR40/phosphoinositide 3-kinase (PI3K) signaling, which was responsible for the stimulation of an atypical protein kinase C (PKC) isoform, PKC*ζ*. Subsequently, AA stimulated phosphorylation of p38 MAPK and transcription factor Sp1, and induced membrane type 3-matrix metalloproteinase (MT3-MMP)-dependent fibronectin degradation in promoting hUCB-MSCs motility. Finally, the silencing of MT3-MMP in AA-stimulated hUCB-MSCs failed to promote the repair of skin wounds owing to impaired cell motility. In conclusion, AA enhances skin wound healing through induction of hUCB-MSCs motility by MT3-MMP-mediated fibronectin degradation, which relies on GPR40-dependent mTORC2 signaling pathways.

Skin wound healing is a dynamic process that involves inflammation, re-epithelization, granulation, vascularization, and tissue remodeling, in which various types of cells migrate into the wound.^1^ Many treatment modalities are applicable to improve skin recovery after injury including cytokines/growth factors and cell-based therapies.^2^ The use of stem cell therapy has been emphasized as a promising adjunct for delayed wound healing.^3^ In this process, the migration of stem cells is an important biological event to exert their beneficial effects which play a key role in cutaneous regeneration.^4, 5^ Although commonly used approaches to manipulating stem cell behaviors include administration of biochemical cocktails and genetic modifications,^6, 7^ these methods are considered impractical for clinical use owing to unexpected potential side effects. In this respect, the important factor for the success of stem cell therapy in cutaneous wounds is the capability to regulate stem cell behaviors with predictable methods. Therefore, the development of safe, effective, and practical means to modulate stem cell migration is a priority in the skin wound-healing field.

Many nutrients including lipid metabolites are receiving tremendous attention as emerging key regulators of stem cell behaviors.^8^ Current research on lipids and lipid metabolites has so far revealed a number of signaling pathways and effector molecules involved in cell functions, such as G-protein-coupled receptor 40 (GPR40),^9^ Src,^10^ mammalian target of rapamycin (mTOR),^11^ and matrix metalloproteinases (MMPs).^10, 12^ Interestingly, these signaling molecules are also closely related to stem cell behaviors.^9, 13, 14^ Thus, the specific effects of lipid metabolites on the regulation of stem cell functions are important in stem cell biology as well as stem cell-based therapy, which might differ by cell type, dosage, culture conditions, and metabolic state. To date, however, despite the fact that arachidonic acid (AA), a representative ω-6 polyunsaturated fatty acid (PUFA), is the second most abundant fatty acid released into a wound site during skin injury^15, 16^ and is implicated in the restoration of various types of tissues including intestine and bone,^17, 18^ the functional role of AA in stem cell motility is not fully understood. Therefore, the complexity of AA-mediated regulatory networks in stem cell migration is both a challenge and an opportunity for further comprehensive investigations into stem cell-based skin wound therapy.

Mesenchymal stem cells (MSCs) have been shown to ameliorate wound healing in several studies.^3^ Human umbilical cord blood-derived mesenchymal stem cells (hUCB-MSCs) are characterized as neonatal stem cells which are abundant, convenient to obtain, capable of multilineage differentiation, and able to self-renew with a high proliferative capacity.^19^ Because hUCB-MSCs also retain a lower immunogenicity and higher migratory ability, their clinical potential has been assessed with cell-based applications for diseases including chronic skin wounds, colitis, and neurotmesis injuries.^20, 21, 22^ Thus, elucidating the role of AA in modulating behaviors of hUCB-MSCs is a promising and practical approach for clinical applications. Therefore, we investigated the role of AA in transplanted hUCB-MSCs for skin wound healing *in vivo* and its molecular mechanism *in vitro*.

## Results

### AA amplified both skin wound-healing capacity and motility of hUCB-MSCs

**Figure 1:**
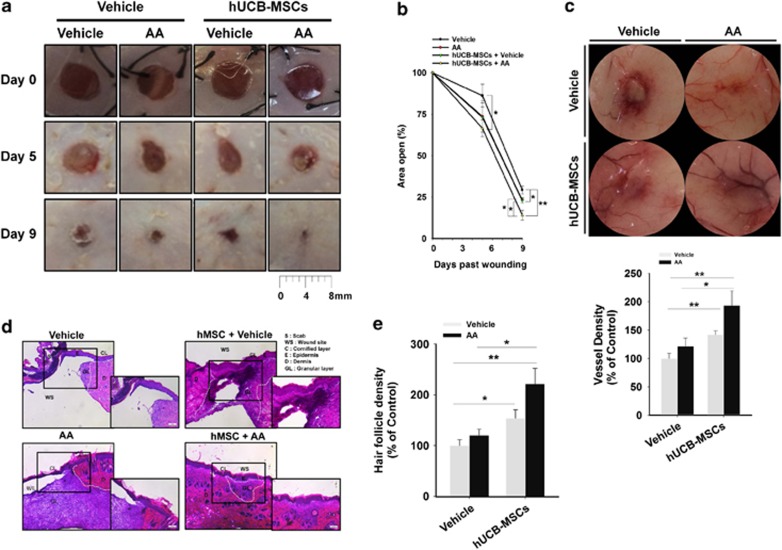
AA enhances skin wound-healing effect of hUCB-MSCs. Two 6-mm wounds were made on the back of each ICR mouse, where we treated vehicle, AA, hUCB-MSCs + vehicle, and hUCB-MSCs + AA by topical application and intradermal injection. (**a**) Representative images of mouse cutaneous wounds on postoperative days are shown. (**b**) Open wound areas relative to the original wound size were quantified by using Image J program. (**c**) Representative images of neovasculature in wounds at day 9. Vessel densities relative to the group treated with vehicle alone were quantified by using Image J program (lower panel). (**d**) Representative H&E sections of wound tissues at day 9 are shown. (**e**) Hair follicle densities relative to the group treated with vehicle alone were quantified. (**a–e**) *n*=5. Error bars represent the mean ±S.E. *P<0.05, **P<0.01. Scale bars=100 *μ*m

In order to evaluate the cutaneous wound-healing effect of hUCB-MSCs and AA, we grossly assessed wound closure and neovascularization in a mouse skin excisional wound model. The group transplanted with hUCB-MSCs pre-treated with AA showed the fastest wound closing (Figures 1a and b) and neovascularization (Figure 1c), while the control group treated with vehicle alone exhibited the slowest wound healing and few vessels. Intriguingly, addition of hUBC-MSCs+vehicle has stimulatory effect on wound closing and neovascularization, compared with the vehicle alone. However, our data revealed there was no statistical difference between AA alone and vehicle in angiogenesis, indicating AA alone did not show a significant enhancing effect on angiogenesis despite of its promoting effect on skin wound closure. This means that the wound healing induced by direct treatement of AA on wound bed is an independent mechanism of the angiogenesis that was initiated from hUBC-MSCs activated by AA. Thus, stem cells primed by AA appear to have specific modes of action that differ from ectopic application of AA in promoting the skin wound healing. Histological evaluation of skin wounds by hematoxylin and eosin (H&E) staining showed that the granulation tissue of wounds treated with AA or hUCB-MSCs did not make intimate contact with the surrounding tissue. In contrast, transplantation of hUCB-MSCs pre-treated with AA induced intimate contact of granulation and wound maturation (Figure 1d). The result of histomorphometric analysis revealed that the group transplanted with hUCB-MSCs pre-treated with AA has more hair follicle density in recovered skin tissue (Figure 1e).

**Figure 2:**
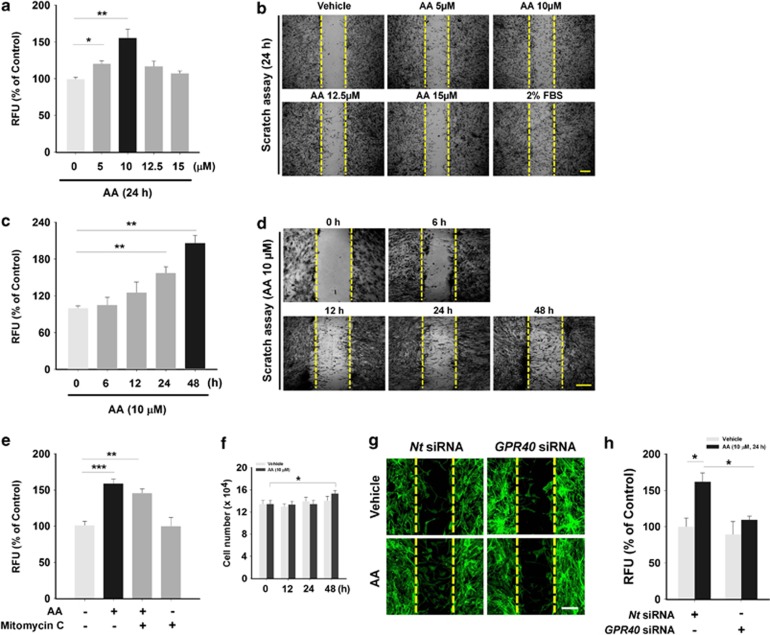
Effect of AA on hUCB-MSCs migration. (**a** and **c**) Dose and time responses of hUCB-MSCs treated with AA in Oris cell migration assay are shown. Cells were incubated with different doses of AA (5–15 *μ*M) for 24 h and with 10 *μ*M of AA for different time periods (0–48 h), respectively. (**b** and **d**) The dose and time responses to AA are also shown in scratch assay. Cells were treated with AA in the same manner as used in Figures 2a and c. Two percent fetal bovine serum was added for positive control. (**e**) The effect of Mitomycin C on AA-induced cell migration was evaluated with Oris cell migration assay. Cells were pretreated with the Mitomycin C (1 *μ*g/ml) for 30 min and then stimulated with 10 *μ*M of AA for 24 h. (**f**) Cells were incubated with 10 *μ*M of AA for 0–48 h and analyzed for their proliferation by cell counting. (**g** and **h**) Cells transfected with *GPR40* specific siRNA (100 nM) were incubated with 10 *μ*M of AA for 24 h and the motility was assessed by (**g**) wound healing assay and (**h**) Oris cell migration assay. (**a–d**) *n*=3. (**e**) *n*=4. (**f**) *n*=6. (**g** and **h**) *n*=3. Data represent means±S.E. *P<0.05, **P<0.01, ***P<0.001. Scale bars=100 *μ*m (magnification × 100). Abbreviations: RFU, relative fluorescence units

For wound restoration, stem cell migration is a prerequisite for recruitment to the wound site.^23^ Thus, we investigated the effect of AA on hUCB-MSCs migration by treatment with various concentrations of AA for 24 h. AA statistically enhanced the cell migration at 5 and 10 *μ*M, but not at higher concentrations (Figure 2a). In addition, treatment with 10 *μ*M of AA for various time periods promoted hUCB-MSCs migration in a time-dependent manner (Figure 2c). The same results were visually confirmed with scratch wound-healing assay (Figures 2b and d). To determine how much the AA-enhanced cell migration solely accounts for covering the mechanically denuded area in the migration assays, proliferation of the cells was evaluated with Mitomycin C, a cell cycle-arresting compound. The cell proliferation was found to increase much less compared with the cell migration (Figure 2e). Although the little effect of AA on hUCB-MSCs proliferation was seen in the result of cell counting (Figure 2f), the results after the [^3^H]thymidine incorporation of hUCB-MSCs revealed that AA did not show any stimulatory effect on the cell proliferation (Supplementary Figure S1). We have performed additional experiments to check whether AA induces the differentiation of hUCB-MSCs *in vitro*. As shown in Supplementary Figure S2, however, the mRNA expression of differentiation markers for osteoblast (Runx2, Osteopontin), adipocyte (PPARγ, FABP4), chondrocyte (Sox9, Col2a1), and endothelial cell (VE-Cadherin, PECAM1) was not significantly regulated by 10 *μ*M of AA treatment for 9  days, suggesting that 10 *μ*M of AA treatment for 9 days seems not to be enough to initiate the differentiation of hUCB-MSCs. Importantly, AA did not have any significant effect on the expression of *COX-1*, *COX-2*, *ALOX5*, *ALOX12*, *ALOX15*, *CYP4A11/22*, and *CYP2J2*, which are major enzymes involved in AA metabolism (Supplementary Figure S3a). Moreover, the cell migration induced by AA was not regulated by pre-treatment with non-selective inhibitors for COX (indomethacin), LOX (NDGA), and CYP (1-ABT) (Supplementary Figure S3b), suggesting that hUCB-MSCs migration is truly attributed by AA itself, but not by AA metabolites. Interestingly, the AA-induced cell migration was abolished by transfection with small-interfering RNA (siRNA) for a membrane receptor for ω-6 PUFAs, GPR40, in an *in vitro* wound-healing migration assay (Figure 2g) and in an Oris cell migration assay (Figure 2h), indicating the involvement of GPR40 in AA-promoted hUCB-MSCs motility.

### AA activates mTORC2, Akt^ser473^, and PKC*ζ* through GPR40/PI3K signaling

**Figure 3:**
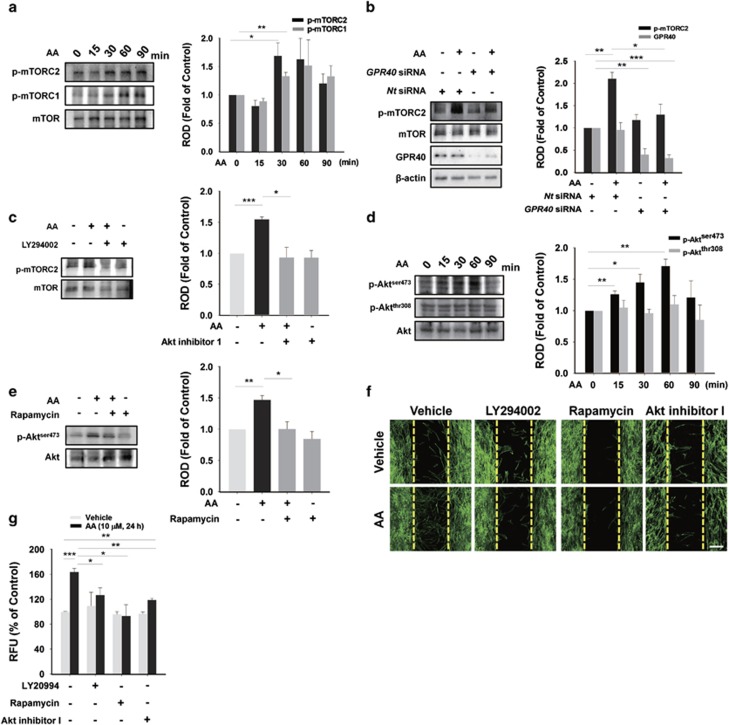
AA promotes phosphorylation of mTOR and Akt^ser473^. (**a** and **d**) hUCB-MSCs were incubated with 10 *μ*M of AA for various time periods (0–90 min). Total proteins were extracted and blotted with mTORC1, phosphor-mTORC2, mTOR, phosphor-Akt^ser473^, phosphor-Akt^thr308^, or Akt. (**b**) Cells transfected with *GPR40* specific siRNA (100 nM) were incubated with 10 *μ*M of AA for 24 h and the levels of phosphor-mTORC2, mTOR, GPR40, and *β*-actin were examined by western blotting with total cell lysates. (**c**) Cells pre-treated with 10 *μ*M of LY294002 for 30 min were incubated with 10 *μ*M of AA for 30 min. Phosphor-mTORC2 and mTOR were detected by western blotting. (**e**) Cells were pre-treated with 10 nM of rapamycin for 12 h prior to AA exposure for 60 min. Phosphor-Akt^thr473^ and Akt were detected by western blotting. Ten nano mole of Rapamycin (for 12 h), 10 *μ*M of LY294002 (for 30 min), and 10 *μ*M of Akt inhibitor I (for 30 min) were pre-treated to cells prior to 10 *μ*M of AA incubation for 24 h. The inhibitory effect on the AA-enhanced hUCB-MSCs migration was examined in wound healing assay (**f**) and Oris cell migration assay (**g**). (**a**–**g**) *n*=3. Data represent means±S.E. *P<0.05, **P<0.01, ***P<0.001. Scale bars=100 *μ*m (magnification × 100). Abbreviations: RFU, relative fluorescence units; ROD, relative optical density

We further examined whether the GPR40/PI3K/mTOR pathway is involved in promoting the motility of hUCB-MSCs. The phosphorylation of mTOR^ser2481^ (mTORC2) induced by AA peaked at 30 min and mTOR^ser2448^ (mTORC1) did at 60 min (Figure 3a). We focused on the early-peaked mTORC2 which are present at upstream signaling network compared with mTORC1.^24^ The AA-induced increase in phosphorylation of mTORC2 was significantly abrogated by transfection with *GPR40* siRNA (Figure 3b) and by pre-treatment with PI3K inhibitor, LY294002 (Figure 3c). We then assessed phosphorylation of Akt closely associated with mTOR signaling. The phosphorylation level of Akt^ser473^ increased until 60 min in contrast to stationary Akt^thr308^ (Figure 3d). In addition, the phosphorylation of Akt^ser473^ was blocked by prolonged rapamycin pre-treatment, which is able to inhibit mTORC2 (Figure 3e).^25^ We also found that AA-induced cell motility was inhibited by LY294002, rapamycin, and Akt inhibitor I in an *in vitro* wound-healing migration assay (Figure 3f) and in an Oris cell migration assay (Figure 3g).

**Figure 4:**
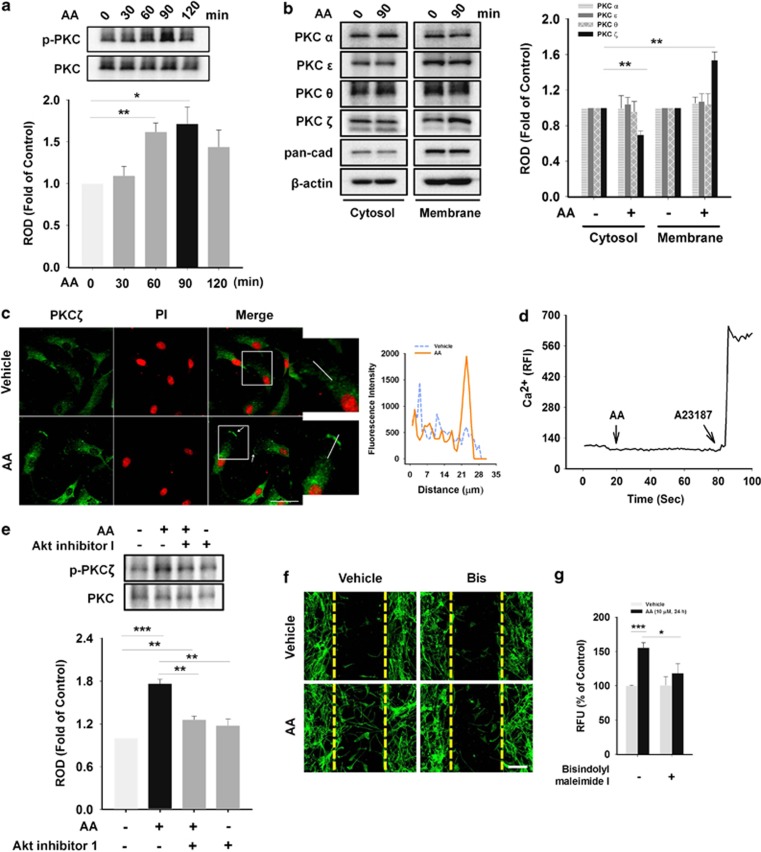
AA stimulates atypical PKC*ζ* translocation. (**a**) hUCB-MSCs were treated with 10 *μ*M of AA for different time periods (0–120 min). Total cell lysates were analyzed by western blotting using phospho-PKC and PKC antibodies. (**b**) Cells were stimulated with 10 *μ*M of AA for 90 min and fractionated into cytosolic and membrane samples. Translocation of PKC isoforms were detected by western blotting and pan-cadherin was used as a control for plasma membrane. (**c**) Cells incubated with 10 *μ*M of AA for 90 min were immunostained with PKC*ζ* antibody (green). PI was used for nuclear counterstaining (red). The arrows point to translocated PKC*ζ* in plasma membrane by AA. And the right graph presents distribution of PKC*ζ* on the yellow lines in the merged images, which were analyzed by FluoView 300 software (Olympus, Tokyo, Japan). Scale bars=100 *μ*m (magnification × 400). (**d**) Cells were loaded with 2 *μ*M of Fluo-3/AM in serum-free medium for 40 min and treated with 10 *μ*M of AA. Ca^2+^ influx was investigated by confocal microscopy and data are expressed as relative fluorescence intensity (F/F0%, arbitrary unit). A23187, a calcium ionophore, was used as a positive control. (**e**) Cells pre-treated with 10 *μ*M of Akt inhibitor I for 30 min were incubated with 10 *μ*M of AA for 90 min. And phospho-PKC*ζ* and PKC were detected by western blotting. In (**f**) wound healing assay and (**g**) Oris cell migration assay, 5 *μ*M of Bisindolylmaleimide I was pretreated to cells for 30 min before treating AA (10 *μ*M) for 24 h, and then their inhibitory effect on the AA-enhanced hUCB-MSCs migration was examined. Scale bars=100 *μ*m (magnification × 100). (**a**, **b**, and **e**–**g**) *n*=3. (**c** and **d**) *n*=4. Data represent means±S.E. *P<0.05, **P<0.01, ***P<0.001. Abbreviations: RFU, relative fluorescence units; ROD, relative optical density

We further checked the involvement of PKC in signaling pathway induced by AA. The phosphorylation of PKC was detected from 60 to 120 min (Figure 4a). Among conventional (PKC*α*), novel (PKC*θ*, *ɛ*), and atypical (PKC*ζ*) PKC isotypes, only atypical PKC*ζ* translocated from the cytosol to the membrane in response to AA treatment (Figure 4b). The membrane translocation was visually confirmed by immunofluorescence staining in AA-treated hUCB-MSCs (Figure 4c). Because atypical PKC do not need calcium for activation, there was no calcium influx in AA-stimulated hUCB-MSCs (Figure 4d). In addition, the PKC*ζ* activation was blocked by pre-treatment with Akt inhibitor I (Figure 4e) and rapamycin (Supplementary Figure S4a). We also found that AA-induced cell motility was inhibited by PKC inhibitor, Bisindolylmaleimide I in an *in vitro* wound-healing migration assay (Figure 4f) and in an Oris cell migration assay (Figure 4g).

### AA promotes membrane type 3 (MT3)-MMP-mediated FN degradation through p38 MAPK/Sp1 cascade

**Figure 5:**
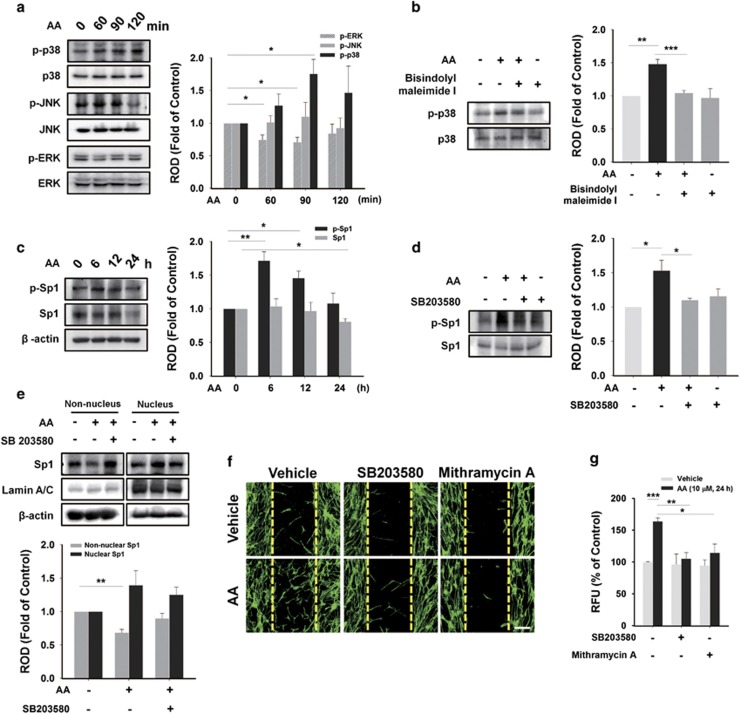
AA-induced phosphorylation of p38 MAPK is involved in Sp1 activation. (**a**) hUCB-MSCs were treated with 10 *μ*M of AA at various time points (0–120 min). Total cell lysates were blotted with phospho-ERK, -JNK, -p38 MAPK, and their total form. (**b**) Cells pre-treated with 5 *μ*M of Bisindolylmaleimide I for 30 min were incubated with 10 *μ*M of AA for 90 min. And phospho-p38 and p38 MAPK were detected by western blotting. (**c**) Cells were exposed to 10 *μ*M of AA for different times (0–24 h), and then total proteins were extracted and examined with phospho-Sp1, Sp1, and *β*-actin antibodies by western blotting. (**d**) Cells were pre-treated with 1 *μ*M of SB203580 for 30 min prior to 10 *μ*M of AA incubation for 6 h. And phospho-Sp1 and Sp1 were detected by western blotting. (**e**) Cells were treated with AA and/or SB203580 (the same manner used in Figure 5d) and fractionated into non-nuclear and nuclear samples. Translocation of Sp1 was blotted, and the Lamin A/C was used as a control for nucleus. In (**f**) wound healing assay and (**g**) Oris cell migration assay 1 *μ*M of SB203580 and 5 *μ*M of Mithramycin A were pretreated to cells for 30 min before treating 10 *μ*M of AA for 24 h, and then their inhibitory effect on the AA-enhanced hUCB-MSCs migration was examined. (**a**–**g**) *n*=3. Data represent means±S.E. *P<0.05, **P<0.01, ***P<0.001. Scale bars=100 *μ*m (magnification × 100). Abbreviations: RFU, relative fluorescence units; ROD, relative optical density

We analyzed the phosphorylation level of MAPKs, which are major PKC substrates.^26^ AA uniquely induced phosphorylation of p38 MAPK from 60 to 120 min (Figure 5a), which was inhibited by pre-treatment with PKC inhibitor, Bisindolylmaleimide I (Figure 5b). But ERK and JNK were not stimulated by AA treatment. We then determined whether AA induces phosphorylation of Sp1, which is a ubiquitous transcription factor that controls gene expression involved in stem cell behavior.^27^ AA induced the phosphorylation of Sp1 from 6 to 12 h (Figure 5c) while no increase was observed before 6 h (data not shown). In addition, the nuclear translocation of Sp1 was observed at 6 h (Figure 5e), and both the phosphorylation and the translocation of Sp1 were blocked by pre-treatment with p38 MAPK inhibitor, SB203580 (Figures 5d and e). Furthermore, pre-treatment with PI3K inhibitor (LY294002) (Supplementary Figure S4b) and mTOR inhibitor (rapamycin) (Supplementary Figure S4c) also blocked the phosphorylation of Sp1 induced by AA. We also found that AA-induced cell motility was inhibited by SB203580 and Sp1 inhibitor, Mithramycin A, in an *in vitro* wound-healing migration assay (Figure 5f) and in an Oris cell migration assay (Figure 5g).

**Figure 6:**
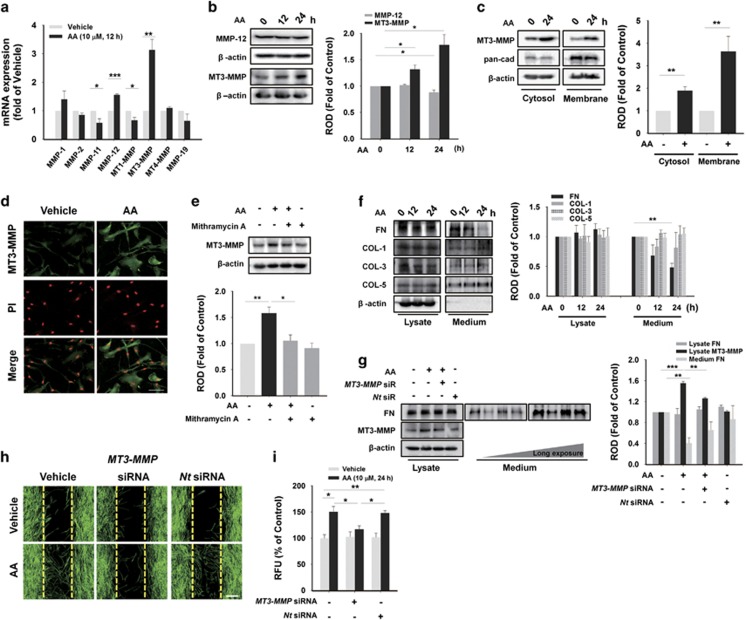
AA stimulates MT3-MMP expression, which degrade FN. (**a**) With real-time PCR, the mRNA expression of *MMP* family was analyzed in hUCB-MSCs treated with 10 *μ*M of AA for 12 h. (**b**) Cells were exposed to 10 *μ*M of AA for different time periods (0–24 h), and total cell lysates were subjected to SDS-PAGE for detecting MMP-12 and MT3-MMP. (**c**) Cells were incubated with 10 *μ*M of AA for 24 h and fractionated into cytosolic and membrane samples. Translocation of MT3-MMP was detected by western blotting, and pan-cadherin was used as a control for plasma membrane. (**d**) Cells treated with 10 *μ*M of AA for 24 h were immunostained with MT3-MMP antibody (green). PI was used for nuclear counterstaining (red). Scale bars=100 *μ*m (magnification × 400). (**e**) Cells were pre-treated with 5 *μ*M of Mithramycin for 30 min prior to 10 *μ*M of AA exposure for 24 h. Then, total proteins were extracted and examined with MT3-MMP antibody by western blotting. (**f**) Cells were treated with 10 *μ*M of AA for 24 h. Western blot assay of FN, COL-1, COL-3, and COL-5 on both cell lysates and proteins precipitated from medium by 30% trichloroethanoic acid was carried out. (**g**) Cells were transfected with *MT3-MMP* siRNA (25 nM) for 24 h prior to 10 *μ*M of AA incubation for 24 h. And protein levels of FN both in cell lysates and in medium were analyzed with western blotting. In (**h**) wound healing assay and (**i**) Oris cell migration assay, *MT3-MMP* specific siRNA (25 nM) were transfected to cells for 24 h prior to 10 *μ*M of AA treatment for 24 h. And then, we examined the inhibitory effect of knockdown of MT3-MMP on the cell migration. Scale bars=100 *μ*m (magnification × 100). (**a**–**c** and **e**–**g**) *n*=3. (**d**) *n*=5. (**h** and **i**) *n*=4. Data represent means±S.E. *P<0.05, **P<0.01, ***P<0.001. Abbreviations: RFU, relative fluorescence units; ROD, relative optical density

As Sp1 is responsible for the transcription of many MMP isotypes, which are crucial for cell migration,^28^ mRNA levels of *MMP*s were analyzed with real-time PCR. Among *MMP* isotypes expressed in hUCB-MSCs,^29^ AA distinctively increased the mRNA levels of *MMP-12* and *MT3-MMP*, and decreased *MMP-11* and *MT1-MMP* (Figure 6a). AA also increased the protein expression of MT3-MMP, but did not alter the protein level of MMP-12 (Figure 6b). Additionally, we observed the increased protein level of MT3-MMP in the both cytosol and membrane with western blotting and immunofluorescence staining (Figures 6c and d). Interestingly, we found that the gelatinolytic activity of MT3-MMP was enhanced by AA treatment (Supplementary Figure S5), suggesting that AA promotes the expression of MT3-MMP as well as its activity in hUCB-MSCs. The upregulation of MT3-MMP was abolished by Mithramycin A, an Sp1 inhibitor (Figure 6e). To determine the effect of MT3-MMP on extracellular matrix (ECM) degradation, we analyzed protein levels of FN and COLs, which are major components of ECM. Under a state of uniform expression of those proteins in whole-cell lysates, AA uniquely induced FN degradation from 12 to 24 h while there were no significant changes in COL−1, −3, and −5 in the medium (Figure 6f). By transfecting hUCB-MSCs with *MT3-MMP* siRNA, we observed the abolishment of AA-induced not just FN degradation, but also cell migration (Figures 6g–i).

### AA-upregulated MT3-MMP stimulates the skin wound-healing effect of hUCB-MSCs

**Figure 7:**
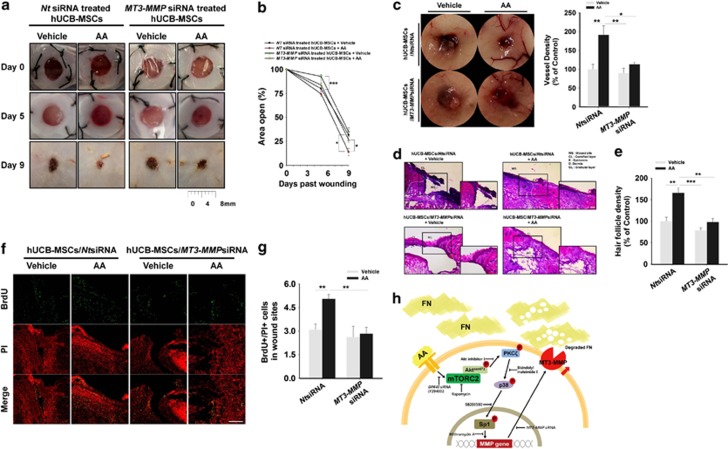
AA-upregulated MT3-MMP affects skin wound healing. Wounds were made with the same manner used in Figure 1 and transplanted with hUCB-MSCs/*MT3-MMP*siRNA (25 nM) or hUCB-MSCs/*Nt*siRNA (25 nM), which were divided into groups pre-treated with AA (10 *μ*M) or vehicle, respectively. (**a**) Representative images of mouse cutaneous wounds on postoperative days are shown. (**b**) Open wound areas relative to the original wound size were quantified with Image J program. (**c**) Representative images of neovasculature in wounds at day 9. Vessel densities relative to the group treated with vehicle alone were quantified with Image J program (right panel). (**d**) Representative H&E sections of wound tissues at day 9 are shown. Scale bars=100 *μ*m (magnification × 100). (**e**) Hair follicle densities relative to the group treated with vehicle were quantified. (**f** and **g**) BrdU-labeled hUCB-MSCs were topically implanted onto the wound bed and injected into the dermis of the surrounding skin. In wound site at day 9, the labeled hUCB-MSCs were determined with confocal microscopy. BrdU was stained with immunofluorescence antibody (green). PI was used for nuclear counterstaining (red). Scale bars=200 *μ*m (magnification × 100). (**h**) A hypothetical model for AA-induced signaling pathway in promoting hUCB-MSCs migration. (**a**–**e**) *n*=5. (**f** and **g**) *n*=4. Data represent means±S.E. *P<0.05, **P<0.01, ***P<0.001. Abbreviations: RFU, relative fluorescence units

In experiments to determine whether AA-upregulated MT3-MMP contributes to the skin wound-healing effect of hUCB-MSCs, the hUCB-MSCs/*non-targetring (Nt)* siRNA induced a better wound-healing effect than that of hUCB-MSCs/*MT3-MMP*siRNA in the mouse model with gross evaluation at day 5 (Figures 7a and b). In addition, hUCB-MSCs/*Nt*siRNA pre-treated with AA had a better effect than with the vehicle up to 20% at day 9 (Figures 7a and b). Furthermore, we found that hUCB-MSCs/*Nt*siRNA pre-treated with AA induced more vessels around the wound, and the knockdown of MT3-MMP resulted in an inhibitory effect on angiogenesis in gross examination of neovasculature (Figure 7c). Histological evaluation of H&E-stained skin wounds showed that all groups (except for the hUCB-MSCs/*Nt*siRNA pre-treated with AA) had imperfect wound closure or granulation which did not make intimate contact with the surrounding tissue (Figure 7d) and had less hair follicle density in recovered wound area (Figure 7e). To evaluate how many transplanted cells migrated to the wound site for restoration, we counted 5-bromo-2'-deoxyuridine (BrdU)-stained hUCB-MSCs at the site and found that the number of the hUCB-MSCs/*Nt*siRNA pre-treated with AA was more than that of the other groups by 70% (Figures 7f and g). In addition, we confirmed the majority of BrdU-labeled cells in wound tissues were not co-localized with endothelial cells marker, CD34, indicating that exogenous hUCB-MSCs did not seem to be differentiated into endothelial cells (Supplementary Figure S6).

## Discussion

In the present study, we demonstrated that AA enhances skin wound healing through the induction of hUCB-MSCs migration in which AA stimulates GPR40 coupling with mTORC2 signaling to regulate MT3-MMP-mediated FN degradation (Figure 7h). We first found that AA by itself has the ability to aid cutaneous wound repair, suggesting that AA might amplify the bioactivity of various migrating cells. Consistently, the transplantation of hUCB-MSCs, pre-treated with AA, was shown to accelerate skin regeneration through tissue re-formation and blood vessel supply. The majority of mesenchymal stem cells has been shown to enhance the angiogenesis *via* the paracrine mechanism rather than the multilineage differentiation.^23^ Thus, it is possible that AA induces motility of hUCB-MSCs to enhance the mobilization and recruitment of stem cells into wound site, where hUCB-MSCs activate paracrine mechanisms to promote vascular growth and angiogenesis.^5, 30^ Therefore, our results suggest that the pre-activation of the hUCB-MSCs with AA could potentiate cell transplantation therapy not only with timely efficacy, but also with reduction of the side effects of overdose of AA. In addition, the pre-activation of UCB-MSCs with AA may offer a means of improving the potency of these cells without the need for additional cell numbers. It should be noted that the proper concentration of AA is critical to improve the outcome of stem cell treatment. In this study, we found that ≤10 *μ*M of AA stimulates hUCB-MSCs migration in a dose- and time-dependent manner, but ≥15 *μ*M of AA gradually decreases the migration. A similar result was also reported in which self-renewal of neural stem/progenitor cells diminished over a specific concentration of AA (10 *μ*M).^31^ These results show that the proper concentration of AA is crucial in stem cell-based therapy and that relatively large amounts of AA are a burden on adult stem/progenitor cells, which is closely associated with oxidative stress from lipid peroxidation.^32^ In this respect, AA levels are not just permissive for stem cell functions but also determinative for at least certain aspects of the functions.

Although the roles of *ω*-6 PUFAs in proliferation and angiogenic effect of stem cells have been well studied,^9, 33^ the functional role of AA by itself in stem cell migration has not been elucidated. GPR40, a membrane receptor for *ω*-6 PUFAs, is known to govern neurogenesis, nutrient sensing in the pancreas, inflammatory condition of the skin, and even proliferation of embryonic stem cells.^9, 34, 35, 36^ In contrast to a previous report showing that some ω-6 PUFAs could regulate cellular events by interacting with cytosolic effectors,^37^ we found that AA directly promotes stem cell migration *via* GPR40, suggesting that the GPR40 activation is the critical requirement in improving the bioactivity of hUCB-MSCs for skin wound healing. mTOR, a pivotal regulator of cell metabolism and behaviors, integrates both extracellular and intracellular signals,^38^ but the relationship between GPR40 and mTOR has not been investigated. In fact, our data revealed that GPR40-dependent activation of the PI3K/mTORC2/Akt^ser473^ pathway has a key role in AA-induced hUCB-MSCs migration. However, the Akt^thr308^/mTORC1 pathway was not involved in this process. These results are consistent with previous reports showing the distinctive role of mTORC1 and mTORC2.^11, 39, 40^ In those reports, the Akt^thr308^/mTORC1 cascade mainly controlled cell growth, whereas cytoskeletal re-organization related to cell motility was critically regulated by the mTORC2/Akt^ser473^ cascade. Moreover, we showed that mTORC2 has the capacity to stimulate mTORC1 through Akt^ser473^ activation. Thus, our results suggest that mTORC2 is not only a major signaling hub in controlling AA-mediated hUCB-MSCs migration, but also a unique AA sensor that receives signals transduced from GPR40. This is further supported by a previous report describing that nutrient/redox/mitogenic input modulates the involvement mTORC2 signaling in cell movement.^41^ To the best of our knowledge, this is the first study to show the relationship between GPR40 and mTOR. Our findings suggest that the physiological activation of mTORC2 may be required for proper skin wound healing and that further activation of other members of the mTORC2 signaling pathway may also be a viable strategy to influence skin recovery.

It was previously documented that lysophosphatidic acid or amino acids induce mTORC2-mediated phosphorylation of Akt^ser473^, PKC*α*, or PKCδ for cell migration.^42, 43^ In contrast, we revealed that AA stimulates atypical (a) PKC*ζ* through mTORC2/Akt^ser473^ activation in enhancing hUCB-MSCs motility. This discrepancy could be explained by that *ω*-6 PUFAs are potent aPKC*ζ* stimulators.^44^ Indeed, *ω*-6 PUFAs were reported to promote oligonucleotide internalization through aPKC*ζ* activation.^45^ Thus, our data show that mTORC2 could transduce AA-induced signals to aPKC*ζ via* Akt^ser473^. Having shown the functional role of aPKC*ζ* is hUCB-MSC migration induced by AA, we further studied the mechanism on how aPKC*ζ* links to other key molecules in stem cell migration. Interestingly, AA through aPKC*ζ* selectively induced p38 MAPK-dependent activation of Sp1, which has the potential ability to modulate cell migration in response to some PUFAs.^46, 47, 48^ However, both ERK and JNK were not stimulated by AA, suggesting a cell-specific role of AA in determining downstream targets. Our results are further supported by a previous report showing that AA regulates both p38 MAPK and ERK for transcriptional activation of the cAMP response element-binding protein (CREB) in driving vascular smooth muscle cell movement.^49^ Although MAPKs have been documented to be capable of controlling the transcriptional activity of Sp1,^50, 51^ the activation of Sp1 occurred long after that of p38 MAPK. This result is probably due to O-glycosylation of Sp1 by p38 MAPK, which contributes to nuclear translocation in which Sp1 is phosphorylated.^27, 52^ Thus, our results indicate that Sp1 has an important role in hUCB-MSCs migration by receiving the unique AA signaling pathway. Here, we show for the first time that the addition of AA had beneficial effects, improving the quality of hMSCs generated from cord blood through AA-induced bioactive signaling molecules.

During wound regeneration, MMPs remodel the ECM, allowing the penetration of stem cells and blood vessels into the wound site for tissue repair.^53, 54^ Although MT-MMPs have been reported to dissolve the basement membrane directly and promote adult stem/progenitor cell motility,^55, 56, 57, 58^ their functional role in stem cell migration and their effects on skin wound healing have not been characterized. In the present study, we found that AA uniquely increases the level of MT3-MMP among all the isoforms of MMP to regulate ECM degradation. In contrast to our previous reports showing that secretory MMP-12 participates in the mobilization of hUCB-MSCs in response to some microenvironmental factors,^14, 29^ we identified that AA exclusively upregulates MT3-MMP through Sp1 in promoting the motility of hUCB-MSCs. This implies that the activation of MMPs is distinctively regulated by microenvironmental factors that affect the transcription of many MMP isotypes. Despite the fact that MT3-MMP could degrade FN and COL-3 among the ECM proteins,^59^ we found that MT3-MMP uniquely degraded FN, which is abundant during the stage of granulation and angiogenesis during wound repair.^60^ In fact, it has been documented that MT3-MMP modifies the pericellular ECM, allowing the cells to have more functional podia highly specialized for migration.^61^ The results highlight that MT3-MMP specifically mediates the motility of hUCB-MSCs induced by AA.

Consistently, we found that the silencing of the MT3-MMP in hUCB-MSCs pre-activated with AA failed to regulate stem cell motility, resulting in a significant delay in *in vivo* angiogenesis and wound healing despite the AA pre-treatment. This means that the phenotypic outcome of hUCB-MSCs induced by AA *in vitro* is well maintained during skin wound healing in mouse. As MT3-MMP is a membrane-bound metalloproteinase and not a secreted one, we thought that MT3-MMP itself does not directly regulate angiogenesis *in vivo*. Instead, MT3-MMP plays a critical role in promoting the motility of hUCB-MSCs. Thus, it is possible that MT3-MMP cleaves pericellular substrate including FN and could therefore allow cells to migrate into wound sites. These mean that AA controls surface restriction of MT3-MMP to specifically degrade FN in promoting the migration of stem cells into wound site where the hUCB-MSCs induce the angiogenesis *via* paracrine mechanism or enhance the skin wound healing with specific modes of action.

Taken together, our findings suggest that pre-activation of hUCB-MSCs would provide new methods for stem cell therapy in wound healing and contribute to rationally develop culture conditions with optimal AA concentration or AA-based intervention protocols that could lead to long-term cost-effective outcomes in hUCB-MSCs-based skin wound therapy. In conclusion, AA promotes skin wound healing through induction of hUCB-MSCs motility for which AA binding to GPR40 stimulates the PI3K/mTORC2/Akt^ser473^/PKC*ζ*/p38 MAPK/Sp1 cascade leading to MT3-MMP-dependent FN degradation.

## Materials and Methods

### Materials

hUCB-MSCs were obtained from MEDIPOST Co. Ltd (Seoul, Korea). Fetal bovine serum was bought from BioWhittaker Inc. (Walkersville, MO, USA). AA, A23187, bisindolylmaleimide I, BrdU, Indomethacin, LY294002, mitomycin C, Nordihydroguaiaretic acid (NDGA), rapamycin, 1-Aminobenzotriazole (1-ABT), and SB203580 were aquired from the Sigma Chemical Company (St Louis, MO, USA). Phospho-Akt^ser473^, phospho-Akt^thr308^, Akt1/2/3, *β*-Actin, collagen1A, collagen3A, collagen5A, fibronectin, phospho-p38, p38, phospho-JNK, JNK, p-ERK1/2, ERK, lamin A/C, MMP-12, pan-cadherin, PKC*α*, PKC*ɛ*, PKCθ, PKC*ζ*, PKC, phospho-PKC*ζ*, and Sp1 antibodies were obtained from Santa Cruz Biotechnology (Santa Cruz, CA, USA). Phospho-PKC, phospho-mTOR^ser2481^ (mTORC2), phospho-mTOR^ser2448^ (mTORC1), and mTOR were purchased from Cell Signaling (Beverly, MA, USA). The Akt inhibitor I was aquired from Calbiochem (La Jolla, CA, USA). The CD34, GPR40, phospho-Sp1, and MT3-MMP antibodies were obtained from Abcam (Cambridge, MA, USA). Mithramycin A was purchased from Tocris (Bristol, UK). Zymogram gels were bought from Novex (San Diego, CA, USA). Horseradish peroxidase-conjugated goat anti-mouse and goat anti-rabbit IgG were obtained from Jackson Immunoresearch (West Grove, PA, USA). All other reagents were used as received and were of the highest purity commercially available.

### Culture of hUCB-MSCs

Briefly, hUCB-MSCs were cultured without a feeder layer and maintained in *α*-minimum essential medium (Thermo, Tewksbury, MA, USA), 10% fetal bovine serum, and 1% penicillin and streptomycin in a humidified 5% CO_2_ incubator at 37 °C. Before experiments, the medium was replaced with the serum-free medium for 24 h. And then, the cells were washed twice with phosphate-buffered saline (PBS) and maintained in a serum-free medium including all supplements and indicated agents.

### Mouse excisional wound splinting model

All animal experiments were performed with the approval of the Institutional Animal Care and Use Committee of Seoul National University (SNU-140123-6) and in accordance with the National Institutes of Health Guidelines for the Care and Use of Laboratory Animals. In addition, four authors are Doctors of Veterinary Medicine with licenses granted from the Ministry of Agriculture and Forestry of Republic of Korea. Eight week-old ICR mice (♂) were used and anesthetized using a 1 : 2 mixture of Xylazine HCl (10 mg/kg, Rompun, Bayer, Germany) and Zoletil (20 mg/kg, Virbac Laboratories, Carros, France) *via* intra-peritoneal injection prior to all surgery. Mouse cutaneous wounding (two 6-mm wounds on the back) and stem cell implantation were carried out as described previously.^62, 63^ Experimental animals to investigate the functional effect of AA on hUCB-MSCs were separated into four groups (five mice per group); wild-type mice received vehicle (group 1) or AA (group 2) without hUCB-MSCs; and mice topically transplanted with hUCB-MSCs which were pre-treated with vehicle (group 3) or AA (group 4). Furthermore, to study the role of MT3-MMP in the wound-healing effect of hUCB-MSCs, mice were divided into four additional groups (five mice per group) where hUCB-MSCs were pre-treated with 2 *μ*M of BrdU for 24 h before transplantation; mice in two groups were transplanted with hUCB-MSCs/*Nt*siRNA that were pre-treated with vehicle (group 5) or AA (group 6); and mice in the other groups were given hUCB-MSCs/*MT3-MMP*siRNA that were pre-treated with vehicle (group 7) or AA (group 8). We injected 1 × 10^6^ hUCB-MSCs in 70 *μ*l PBS containing 50% growth factor–reduced Matrigel (BD Biosciences, Franklin Lakes, NJ, USA) into the dermis at two sites around the wound and also topically spread 0.3 × 10^6^  hUCB-MSCs in 30 *μ*l PBS containing the same Matrigel onto the wound bed at day 0 and 5. After that, we put Tegaderm (3M, London, ON, Canada) around the wounds. Images of wounds were taken on days 0, 5, and 9 with a digital camera system (D50, Nikon, Tokyo, Japan) at the same camera/subject distance (30 cm). The open areas of wounds at days 5 and 9 were measured using Image J program (NIH, Bethesda, MD, USA) and presented as percentage of the original wound size. The images of inner side of cutaneous wound sites were obtained to evaluate angiogenesis at day 9. The wound tissues were then embedded in O.C.T. compound (Sakura Finetek, Trannce, CA, USA), stored at −70 °C, cut into 6*-μ*m-thick frozen sections by using cryosectioning machine, and mounted on SuperFrost Plus slides (Thermo Fisher Scientific, Rockford, IL, USA) for H&E staining and immunohistochemistry. For histomorphometric analysis, the recovered skin regions adjacent to granulation tissue were evaluated at × 100 magnification in H&E-stained sections. Hair follicles were counted and the relative hair follicle densities were presented as percentage of the group treated with vehicle alone or hUCB-MSCs/*Nt*siRNA+Vehicle.

### Wound-healing migration assay

hUCB-MSCs (4 × 10^4^ cells) were seeded on low 35-mm dishes with silicone inserts (Ibidi, Martinsried, Germany) and cultured until the cells reach around 100% confluence in serum-containing medium. After serum starvation for 24 h, the inserts were removed to create a wound field. The cells were incubated additionally for 24 h with 10 *μ*M of AA and visualized with an Olympus FluoView 300 confocal microscope (Tokyo, Japan) with a × 100 objective.

### Oris cell migration assay

hUCB-MSCs (3 × 10^2^ cells)/100 *μ*l were seeded in each well of Oris plate (Platypus Technologies, Madison, WI, USA) and incubated for at least 24 h to permit cell adhesion. The cells were cultured until they reach around 70% confluence. After that, inserts were carefully removed and the cells were gently washed with warm PBS. The cells were incubated with 10 *μ*M of AA in serum-free medium for 24 h and then treated with 5 *μ*M of calcein AM for 30 min to stain the cell populations in endpoint assays. By using a microplate reader to measure excitation/emission wavelengths (485/515 nm), migrated cells were quantified.

### Scratch wound-healing assay

hUCB-MSCs were cultured until 90% confluence in 35-mm dishes and scratched with a sterile cell scraper (Fisher Scientific, Pittsburgh, PA, USA). The border of the denuded area was marked with a fine line immediately, and the cells were incubated with 10 *μ*M of AA in serum-free medium. Fetal bovine serum (2%) was treated for positive control. The cell migration was observed with microscope during incubation with a × 100 objective.

### Western blot analysis

Cell lysates were extracted with lysis buffer (20 mM Tris (pH 7.5), 1 mM EDTA, 1 mM EGTA, 1% Triton X-100, 1 mg/ml aprotinin, and 1 mM phenylmethylsulfonylfluoride) for 30 min on ice. The lysates were cleared by centrifugation (15 000 r.p.m. at 4 °C for 30 min), and then the protein concentration was determined by the Bradford method.^64^ Equal amounts of protein (15 *μ*g) were resolved by 8–12% SDS-PAGE and transferred to PVDF membranes. The membranes were blocked with TBST solution (10 mM Tris-HCl (pH 7.6), 150 mM NaCl, and 0.05% Tween-20) containing 5% skim milk for 1 h, and incubated with appropriate primary antibodies at 4 °C for overnight. The membrane was then washed and detected with a horseradish peroxidase-conjugated secondary antibody. The membrane were visualized by enhanced chemiluminescence (Amersham Pharmacia Biotech Inc., Buckinghamshire, UK).

### Subcellular fractionation

Harvested cells were mixed with buffer 1 (250 mM sucrose, 50 mM Tris-HCl, 5 mM MgCl_2_) containing protease inhibitor cocktail (PIERCE, Rockford, IL, USA), incubated on an end-over-end shaker for 10 min, and centrifuged at 1000 × *g* for 10 min. The supernatants containing cytosolic proteins were transferred to iced tubes. The pellets were lysed with buffer 2 (1M sucrose, 50 mM Tris-HCl, 5 mM MgCl_2_) containing protease inhibitor cocktail for 30 min and were centrifuged at 6000 × *g* for 10 min. The supernatants with membrane proteins were transferred to new iced tubes. The remaining pellets were suspended in buffer 3 (20 mM Tris-HCl, 0.4M NaCl, 15% glycerol, 1.5% Triton X-100) containing protease inhibitor cocktail and incubated on an end-over-end shaker for 10 min. After centrifugation at 6800 × *g* for 10 min, the supernatants containing nuclear proteins were collected.

### Trichloroethanoic acid precipitation

Filtered culture supernatants were mixed with 100% trichloroethanoic acid (w/v) to a final concentration of 30% (w/v) and were incubated overnight at 4 °C. Samples were centrifuged at 10 000 × *g* for 20 min, and then pellets were washed with cold acetone before air-drying. The protein pellets were dissolved by the same volume of sample loading buffer (40 *μ*l) and subjected to SDS-PAGE.

### Confocal microscopy

Either frozen sections of the wounds or cultured hUCB-MSCs were fixed with 4% paraformaldehyde in PBS for 10 min, permeabilized with 0.2% Triton X-100 in PBS for 5 min, and blocked with 5% normal goat serum in PBS for 30 min at room temperature. And then, the samples were incubated with appropriate primary antibodies for overnight at 4 °C. After washing three times with PBS, the samples were stained with Alexa 488-conjugated goat anti-mouse/rabbit IgM, phalloidin, or BrdU in PBS containing 1% (v/v) BSA. Also, the samples were counterstained with PI at the same time, followed by three times of washing for 10 min with PBS. The samples were visualized with an Olympus FluoView 300 confocal microscope with × 100–400 objective.

### Measurement of calcium influx

Changes in [Ca^2+^]_i_ were measured using Fluo-3-AM (Invitrogen Co., Carlsbad, CA, USA). Cells in confocal 35 mm-diameter coverglass bottom dishes were washed with a Bath solution (140 mM NaCl, 5 mM KCl, 1 mM CaCl_2_, 0.5 mM MgCl_2_, 10 mM glucose, 5.5 mM HEPES, pH 7.4), incubated in a Bath solution containing 2 mM Fluo-3-AM for 40 min, rinsed, and scanned every second with confocal microscope (Fluoview 300, Olympus, Hamburg, Germany) at excitation/emission wavelengths of 488/515 nm. A23187, a calcium ionophore, was used as a positive control. All analyses of [Ca^2+^]_i_ were processed in a single cell, and the results were expressed as the relative fluorescent intensity (RFI).

### RNA isolation and real-time polymerase chain reaction (PCR)

By using the RNeasy Plus Mini Kit (Qiagen, Valencia, CA, USA), total RNA was obtained from hUCB-MSCs. RNA was used for reverse transcription to synthesize cDNA using a Maxime RT premix kit of RNA (iNtRON Biotechnology, Sungnam, Korea). According to the manufacturer's instructions with minor modifications as previously described,^65^ real-time quantification of MMP isotypes was carried out with a QuantiMix SYBR Kit (PhileKorea Technology, Daejeon, Korea) using a Rotor-Gene 6000 real time thermal cycling system (Corbett Research, New South Wales, Australia). GAPDH was used as an endogenous control. The primers used in the present study are described in Supplementary Table S1 or in our previous report.^29^

### Small-interfering RNA transfection

hUCB-MSCs were grown until 70% confluence. The cells were transfected with *MT3-MMP (*25 nM), *GPR40* (100 nM), or *non-targeting* (*Nt*) siRNA (25 or 100 nM) (Dharmacon, Lafayette, CO, USA) using HiPerFect Transfection Reagent (Qiagen, Valencia, CA, USA) for 24 h according to the manufacturer's instructions. The sequences of *GPR40* siRNA were 5′-CGCUCAACGUCCUGGCCAU-3′, 5′-GUGACCGGUUACUUGGGAA-3′, and their complement. The sequences of *MT3-MMP* siRNA were 5′- ACAGGGUGAUGGAUGGAUA-3′, 5′-CAAUGUGGAGGUUUGGUUA-3′, and their complement. The sequences of *Nt* siRNA were 5′- UAGCGACUAAACACAUCAA-3′ and its complement. The siRNA knockdown of *MT3-MMP* efficiently sustained for 5 days (Supplementary Figure S7a) and did not show any effect on the cell viability (Supplementary Figure S7b).

### Cell counting

hUCB-MSCs (4 × 10^5^ cells) were seeded per well and cultured until they reach around 70% confluence. After serum starvation for 24 h, cells were incubated with 10 *μ*M of AA up to 48 h, and the number of cells was counted at different time points using a hemocytometer.

### [^3^H]-thymidine incorporation assay

hUCB-MSCs were synchronized by culture in serum-free media for 24 h and then incubated with 10 *μ*M of AA for 0–48 h. After the incubation period, 1 *μ*Ci of [methyl-^3^H]-thymidine (specific activity: 74 GBq/mmol, 2.0 Ci/mmol; Amersham Biosciences, Buckinghamshire, UK) was added to the cultures for 1 h at 37 °C. Cellular [^3^H]-thymidine uptake was quantified by liquid scintillation counting (Wallac, Turku, Finland) in harvested cellular material. All values were converted from absolute counts to percentages of control.

### Gelatin zymography

Whole-cell lysate samples were resolved under non-reducing conditions on 10% SDS-PAGE gels embedded with 1 mg/ml gelatin. Gels were rinsed for 30 min each in buffer 1 (50 mM Tris, 2.5% Triton X-100, pH 7.5) and buffer 2 (50 mM Tris, 5 mM CaCl_2_, 1 *μ*M ZnCl_2_, 2.5% Triton X-100, pH 7.5), and then incubated in buffer 3 (50 mM Tris, 5 mM CaCl_2_, 1 *μ*M ZnCl_2_, pH 7.5) for 12 h at 37 °C. The gels were stained with Coomassie Blue, and areas of gelatinolytic were visualized as transparent bands.

### MTT assay

Cell viability was assessed by using the conversion of 3-(4,5-dimethyl thiazolyl-2)-2,5-diphenyl tetrazolium bromide (MTT) to formazan *via* mitochondrial oxidation. A total of 10 *μ*l MTT solution was added to each well for 2 h. The mediums were then removed and cells were incubated with 150 *μ*l of DMSO for 30 min. Absorbance at 570 nm was recorded using a spectrophotometer.

### Statistical analysis

All results are expressed as mean value±standard errors (S.E.). All experiments were analyzed by ANOVA, and some experiments were evaluated by comparing treatment means to the control using the Bonferroni-Dunn test. Difference at *P*<0.05 was considered statistically significant.

## Abbreviations

AA: arachidonic acid
COL: collagen
FN: fibronectin
GPR40: G-protein-coupled receptor 40
hUCB-MSCs: human umbilical cord blood-derived mesenchymal stem cells
mTORC: mammalian target of rapamycin complex
MAPK: mitogen-activated protein kinase
MT3-MMP: membrane type 3-matrix metalloproteinase
PKC: protein kinase C
PI3K: phosphoinositide 3-kinase
PUFA: polyunsaturated fatty acid
siRNA: small-interfering ribonucleic acid
