# Supplementary material for: Arachidonic acid promotes skin wound healing through induction of human MSC migration by MT3-MMP-mediated fibronectin degradation
Source: Cell Death Dis. 2015 May 7;6(5):e1750–. doi: 10.1038/cddis.2015.114 (PMC4669694; doi:10.1038/cddis.2015.114)
Supplement: Supplementary Figure S1 [file cddis2015114x1.docx]

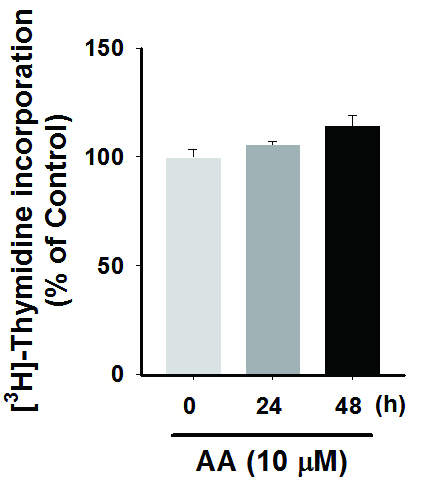


**Supplementary Figure S1. Effect of AA on [^3^H]-thymidine incorporation.** hUCB-MSCs were synchronized by serum starvation for 24 h and then treated with 10 μM of AA for 48 h. [^3^H]-thymidine incorporation was determined. n = 3. Data represent means ± SE.
