# Supplementary material for: Arachidonic acid promotes skin wound healing through induction of human MSC migration by MT3-MMP-mediated fibronectin degradation
Source: Cell Death Dis. 2015 May 7;6(5):e1750–. doi: 10.1038/cddis.2015.114 (PMC4669694; doi:10.1038/cddis.2015.114)
Supplement: Supplementary Figure S2 [file cddis2015114x2.docx]

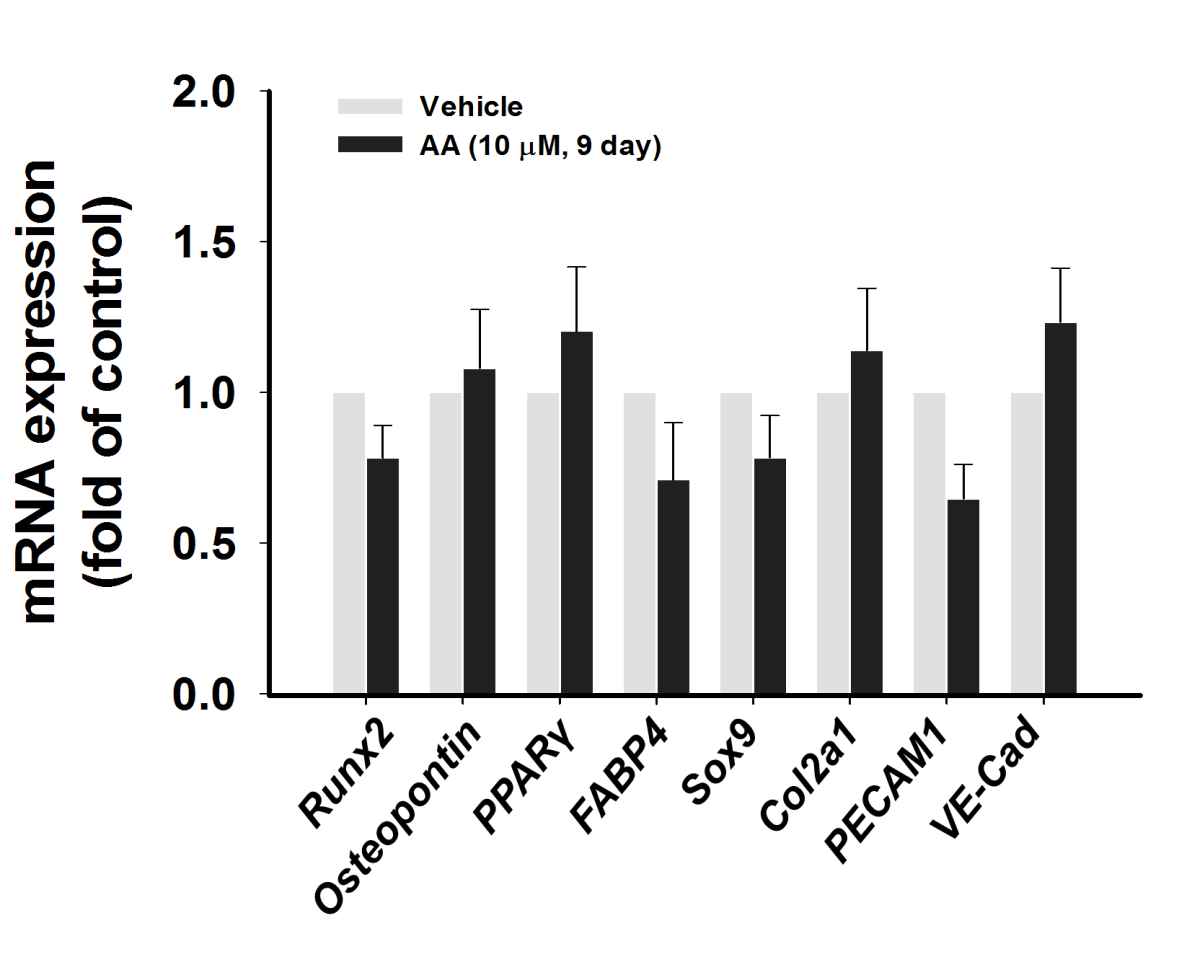


**Supplementary Figure S2. The role of AA in differentiation of hUCB-MSCs.** hUCB-MSCs were incubated with 10 μM of AA for 9 days. The mRNA expression of differentiation makers for osteoblast (*Runx2*, *Osteopontin*), adipocyte (*PPARγ*, *FABP4*), chondrocyte (*Sox9*, *Col2a1*), and endothelial cell (*VE-Cadherin*, *PECAM1*) was assessed by using real-time PCR. n = 3. Data represent means ± SE.
