# Supplementary material for: Arachidonic acid promotes skin wound healing through induction of human MSC migration by MT3-MMP-mediated fibronectin degradation
Source: Cell Death Dis. 2015 May 7;6(5):e1750–. doi: 10.1038/cddis.2015.114 (PMC4669694; doi:10.1038/cddis.2015.114)
Supplement: Supplementary Figure S3 [file cddis2015114x3.docx]

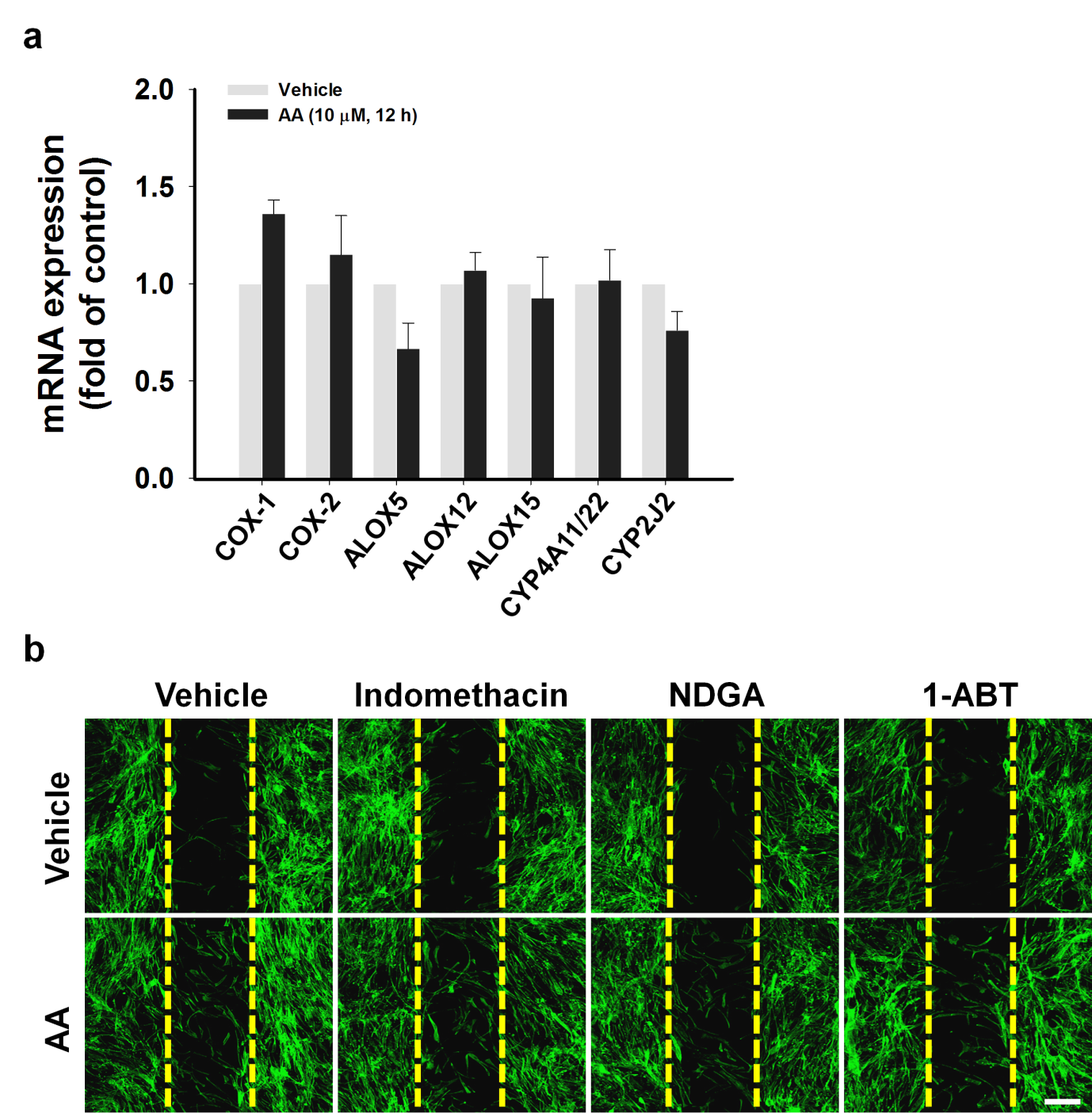


**Supplementary Figure S3. AA metabolism does not regulate the motility of hUCB-MSCs. (a)** hUCB-MSCs were treated with 10 μM of AA for 12 h. The mRNA expression of *COX-1, COX-2, ALOX5, ALOX12, ALOX15, CYP4A11/22*, and *CYP2J2* was measured by using real-time PCR as described in Materials and Methods. Data represent the means ± SE. n=3. **(b)** hUCB-MSCs were pre-treated with indomethacin (10 μM), NDGA (10 μM), and 1-ABT (10 μM) for 30 min prior to AA (10 μM) exposure for 24 h. Wound-healing assay was performed. n=3. Scale bars represent 200 μm.
