# Supplementary material for: Arachidonic acid promotes skin wound healing through induction of human MSC migration by MT3-MMP-mediated fibronectin degradation
Source: Cell Death Dis. 2015 May 7;6(5):e1750–. doi: 10.1038/cddis.2015.114 (PMC4669694; doi:10.1038/cddis.2015.114)
Supplement: Supplementary Figure S4 [file cddis2015114x4.docx]

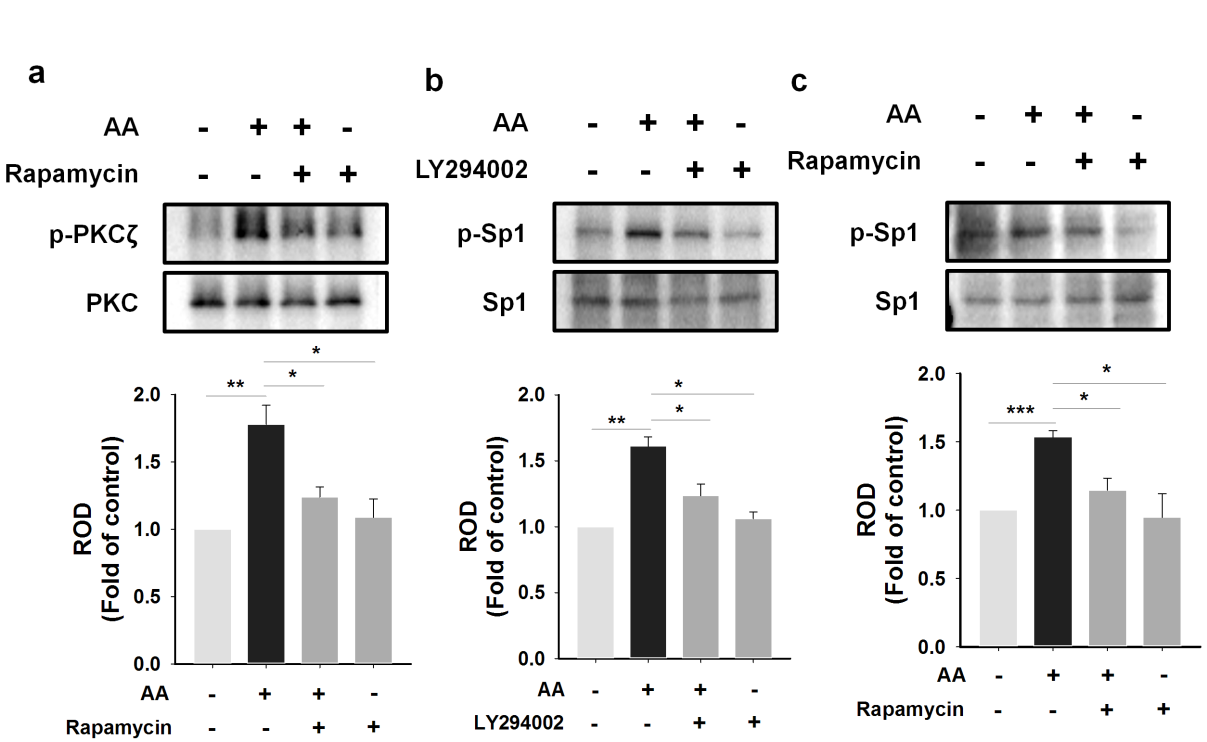


**Supplementary Figure S4. The activation of PIK3 and mTORC2 is responsible for phosphorylation of PKC and SP-1 in hUCB-MSCs treated with AA.** The cells were pretreated with Rapamycin or LY294002 prior to AA (10 μM) incubation. Phosphorylation of PKCζ **(a)** and Sp-1 **(b-c)** was detected by Western blotting. n = 3. Data represent means ± SE. **P* < 0.05, ***P* < 0.01, ****P* < 0.001. Abbreviations: ROD, relative optical density.
