# Supplementary material for: Arachidonic acid promotes skin wound healing through induction of human MSC migration by MT3-MMP-mediated fibronectin degradation
Source: Cell Death Dis. 2015 May 7;6(5):e1750–. doi: 10.1038/cddis.2015.114 (PMC4669694; doi:10.1038/cddis.2015.114)
Supplement: Supplementary Figure S5 [file cddis2015114x5.docx]

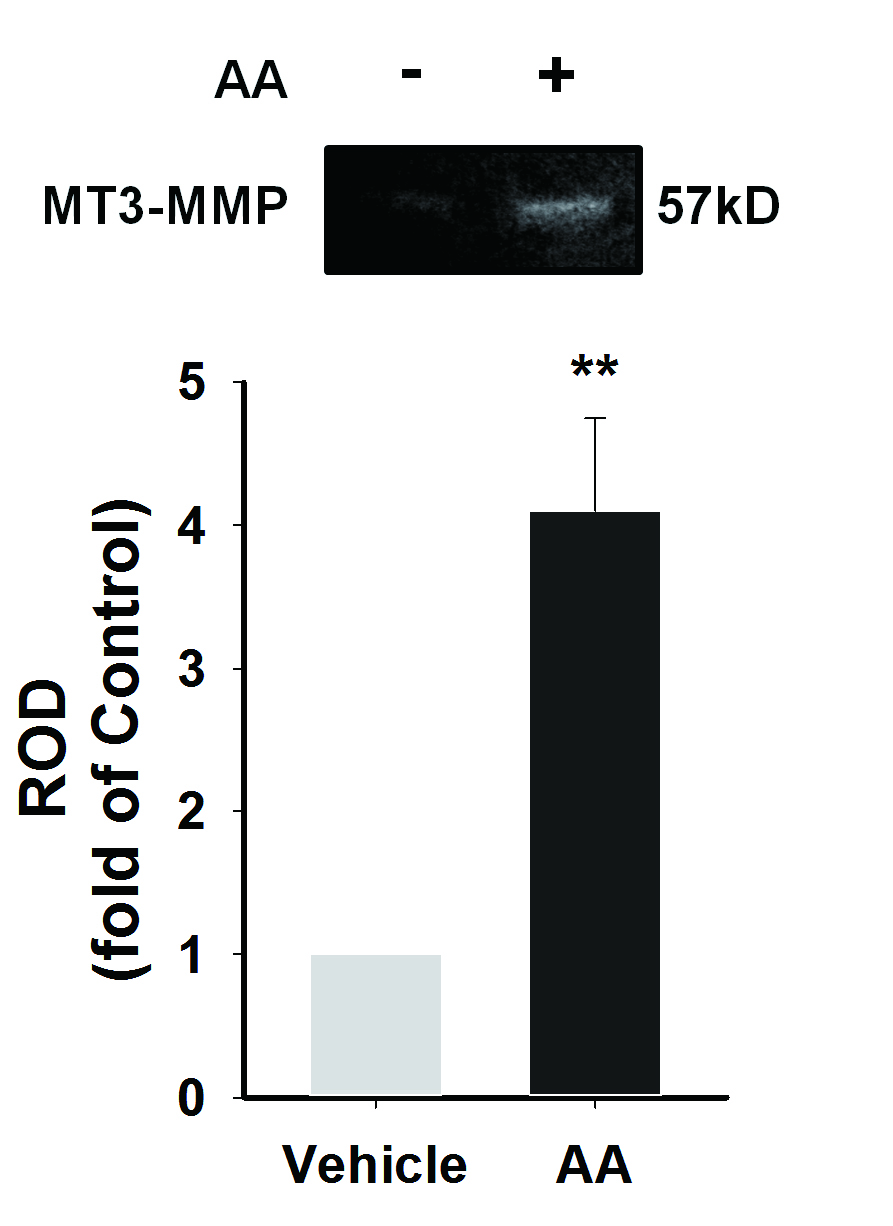


**Supplementary Figure S5. AA enhances enzymatical activity of MT3-MMP in hUCB-MSCs.** hUCB-MSCs were treated with 10 μM of AA for 24 h. Enzymatical activity of MT3-MMP was examined by using gelatin zymography. Data represent means ± SE. n = 3. ***P* < 0.01 versus Vehicle. Abbreviations: ROD, relative optical density.
