# Supplementary material for: Arachidonic acid promotes skin wound healing through induction of human MSC migration by MT3-MMP-mediated fibronectin degradation
Source: Cell Death Dis. 2015 May 7;6(5):e1750–. doi: 10.1038/cddis.2015.114 (PMC4669694; doi:10.1038/cddis.2015.114)
Supplement: Supplementary Figure S6 [file cddis2015114x6.docx]

**
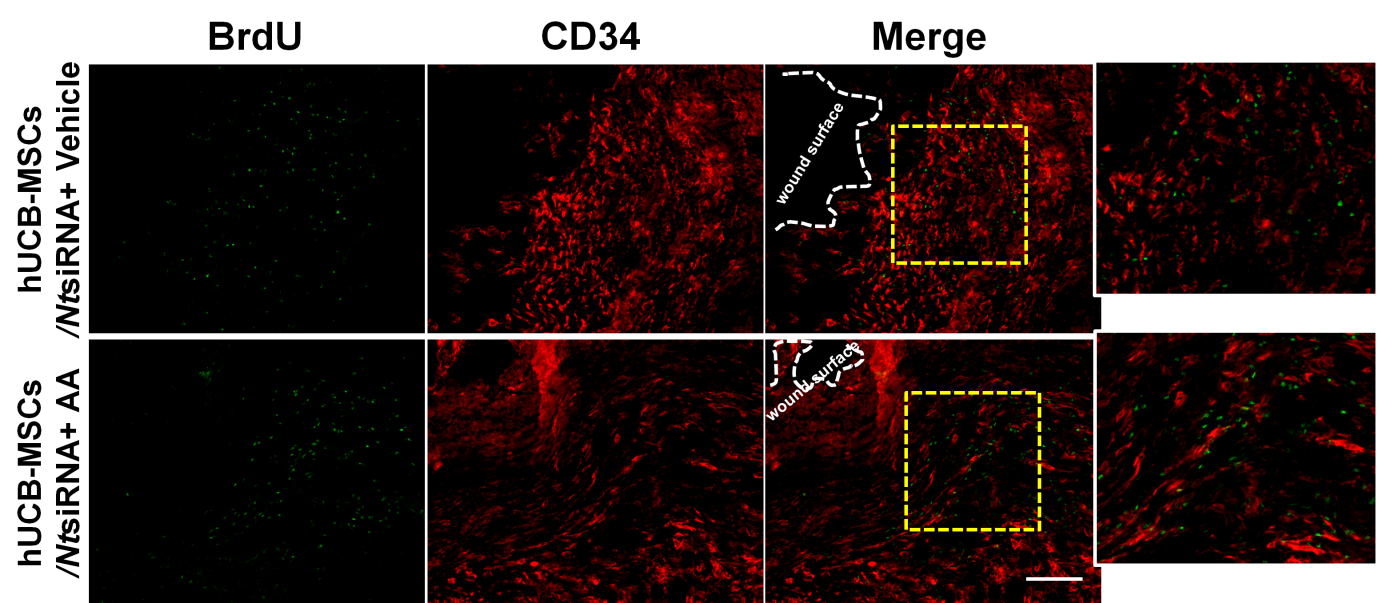
**

**Supplementary Figure S6. Differentiation of hUCB-MSCs pretreated with AA in mouse skin wound healing model.** BrdU-horboring hUCB-MSCs pretreated with AA were topically implanted onto the wound bed and/or injected into the dermis of the surrounding skin. Engraftment of hUCB-MSCs on wound site at day 9 was determined by confocal microscopy using immunofluorescence staining of BrdU (green). CD34 was used for the endothelial cell marker (red). Scale bars represent 100 μm. n = 5. The majority of BrdU-labeled hUCB-MSCs were not co-localized with the CD34.
