# Supplementary material for: Arachidonic acid promotes skin wound healing through induction of human MSC migration by MT3-MMP-mediated fibronectin degradation
Source: Cell Death Dis. 2015 May 7;6(5):e1750–. doi: 10.1038/cddis.2015.114 (PMC4669694; doi:10.1038/cddis.2015.114)
Supplement: Supplementary Figure S7 [file cddis2015114x7.docx]

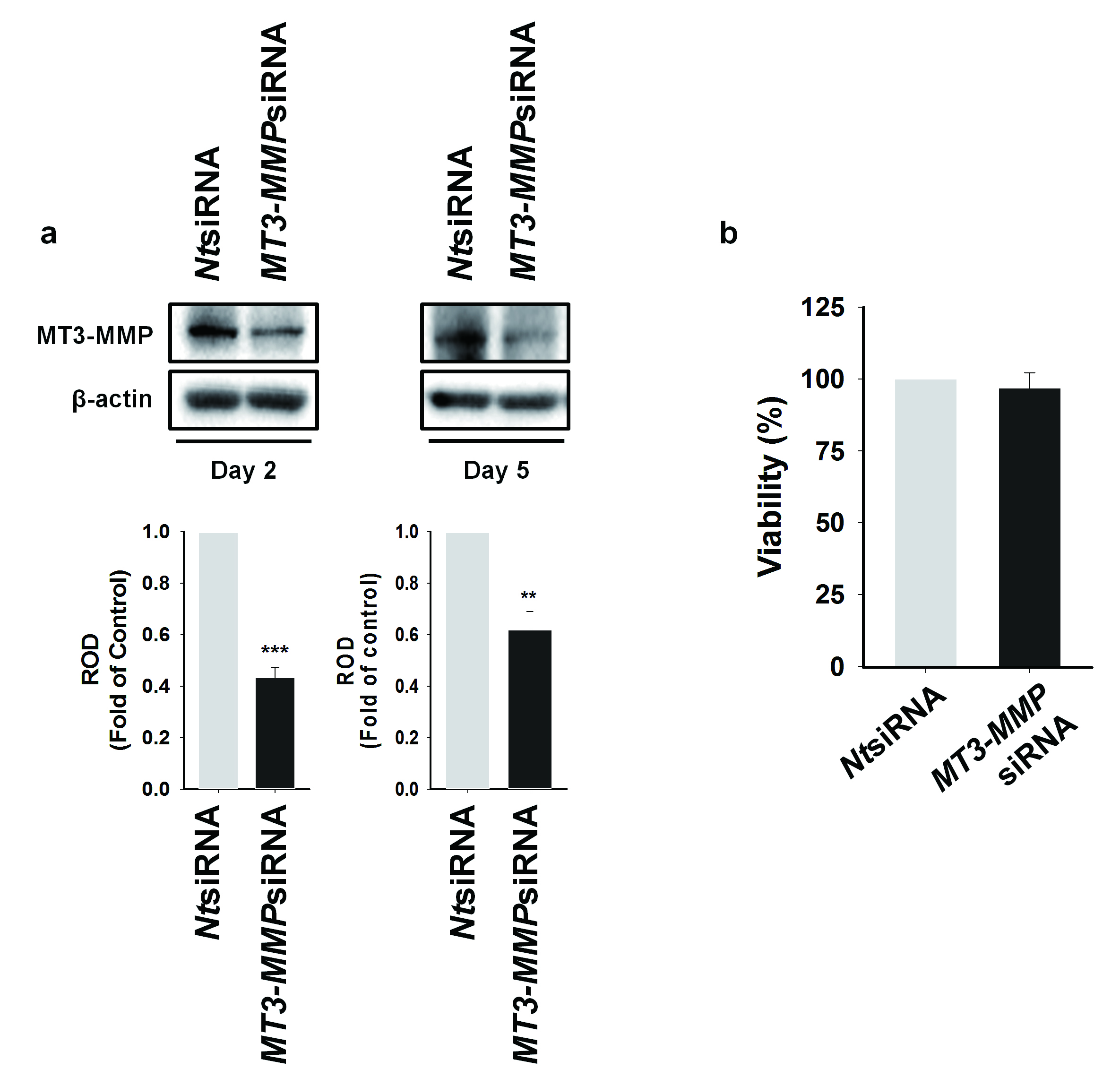


**Supplementary Figure S7. The knockdown efficacy of MT3-MMP siRNA and its effect on cell viability.** Cells were transfected for 24 h with *MT3-MMP* and *non-targeting* (*Nt*) siRNA using HiperFect Transfection Reagent. (**a**) Protein expressions were analyzed by using Western blot. The knockdown efficacy of MT3-MMP was 57% at day 2 and 40 % at day 5. (**b**) The effect of knockdown of MT3-MMP on cell viability was determined by MTT assay. (**a, b**) n = 3. Data represent means ± SE. ***P* < 0.01 versus *Nt*siRNA, ****P* < 0.001 versus *Nt*siRNA. Abbreviations: ROD, relative optical density.
