# Supplementary material for: Arachidonic acid promotes skin wound healing through induction of human MSC migration by MT3-MMP-mediated fibronectin degradation
Source: Cell Death Dis. 2015 May 7;6(5):e1750–. doi: 10.1038/cddis.2015.114 (PMC4669694; doi:10.1038/cddis.2015.114)
Supplement: Supplementary Table S1 [file cddis2015114x8.docx]

**Supplementary Table S1. Primers used for polymerase chain reaction.**

| **Gene** | **Identification** | **Primer sequence, 5’ → 3’** |
| --- | --- | --- |
| ***Runx2*** | Sense | TGGTTAATCTCCGCAGGTCAC |
|  | Antisense | ACTGTGCTGAAGAGGCTGTTTG |
| ***Osteopontin*** | Sense | GCCGAGGTGATAGTGTGGTT |
|  | Antisense | AACGGGGATGGCCTTGTATG |
| ***PPARγ*** | Sense | ACCATGGTGGGTTCTCTCTG |
|  | Antisense | TCAAAGGAGTGGGAGTGGTC |
| ***FABP4*** | Sense | CGTGGAAGTGACGCCTTTCATG |
|  | Antisense | ACTGGGCCAGGAATTTGACGAA |
| ***Sox9*** | Sense | AGTCGGTGAAGAACGGGCA |
|  | Antisense | AAGTCGATAGGGGGCTGTCTG |
| ***Col2a1*** | Sense | CAACACTGCCAACGTCCAGA |
|  | Antisense | CTGCTTCGTCCAGATAGGCA |
| ***VE-Cadherin*** | Sense | ATGACAATGCCCCGGAGTTT |
|  | Antisense | TGTTGGCCGTGTTATCGTGA |
| ***PECAM1*** | Sense | GGACCCTCGTGGATGTTGTA |
|  | Antisense | CTGCTCGGTTCTCTCTGTGA |
| ***Cox-1*** | Sense | TGTGTTGATGCACTACCCCC |
|  | Antisense | TTGTGCTCACGTAGCCAGAG |
| ***Cox-2*** | Sense | GGCCATGGGGTGGACTTAAA |
|  | Antisense | CCCCACAGCAAACCGTAGAT |
| ***ALOX5*** | Sense | AAGGGCGTGGTGACCATTGAG |
|  | Antisense | CGAGGTTCTTGCGGAATCGG |
| ***ALOX12*** | Sense | TCTCTATGCCCATGATGCTTTACG |
|  | Antisense | GGTGAGGAAATGGCAGAGTTGAC |
| ***ALOX15*** | Sense | ACACTTGATGGCTGAGGTCATTG |
|  | Antisense | GGTCGAAAATTCCCATGTCAGAG |
| ***CYP4A11/22*** | Sense | CTTCTGCTGCTGATCAAGGC |
|  | Antisense | AGGACAGGCACTTGGGAATG |
| ***CYP2J2*** | Sense | CTGCGATGGGCTCTGCTTTA |
|  | Antisense | TGCACCTCATGGATGACAGC |
